# Gender Differences in Knowledge and Attitude towards HPV and HPV Vaccine among College Students in Wenzhou, China

**DOI:** 10.3390/vaccines10010010

**Published:** 2021-12-22

**Authors:** Gang Chen, Biao Wu, Xuchao Dai, Mengqi Zhang, Yupeng Liu, Hong Huang, Kun Mei, Zhigang Wu

**Affiliations:** 1 School of Public Health and Management, Wenzhou Medical University, Wenzhou 325035, China; chengang718@163.com (G.C.); XuchaoD1@163.com (X.D.); zhangmengqi324@163.com (M.Z.); liuyupeng@wmu.edu.cn (Y.L.); Huanghongpanda@163.com (H.H.); 2 School of Public Health, Fudan University, Shanghai 200032, China; wubiaozj@163.com; 3 Center for Health Assessment, Wenzhou Medical University, Wenzhou 325035, China; 4 Zhejiang Provincial Key Laboratory of Watershed Science and Health, Wenzhou Medical University, Wenzhou 325035, China; 5 School of Geography Science and Geomatics Engineering, Suzhou University of Science and Technology, Suzhou 215009, China; 6 Department of Urology, The First Affiliated Hospital of Wenzhou Medical University, Wenzhou 325035, China; 7 Reproductive Health Research Center, Health Assessment Center of Wenzhou Medical University, Wenzhou 325000, China

**Keywords:** human papillomavirus, HPV vaccine, college students, knowledge, survey

## Abstract

*Objective*: This study aimed to determine human papillomavirus (HPV)-related awareness and willingness to receive HPV vaccination among college students, in Wenzhou, and its associated factors. *Methods*: A cross-sectional epidemiological study was conducted among college students in Wenzhou to investigate their knowledge, attitude, and factors affecting their willingness to receive HPV vaccination. *Results*: A total of 1035 questionnaires were collected, of which 1002 were valid (males: 374, females: 628). In total, 904 (90.2%) college students had heard of HPV, with a lower rate among males than females (85.3% vs. 93.2%, *p* < 0.05) and 693 (69.2%) had heard of the HPV vaccine, with a significantly lower rate among males than females (53.7% vs. 78.3%, *p* < 0.05). Overall awareness of HPV and HPV vaccine among males and females was moderate, with lower awareness among males. A total of 55.9% of males and 80.4% of females indicated that they would be willing to receive the HPV vaccine, a significant difference (*p* < 0.001). The price, safety of HPV vaccine, and lack of knowledge about HPV and HPV vaccine were the major barriers to HPV vaccination for college students. Compared to females, inadequate knowledge of HPV was the main barrier factor for HPV vaccination among male college students. *Conclusions*: The overall knowledge level of males is lower than that of females. For male college students, providing more knowledge about HPV infection is helpful to promote their willingness to vaccinate. It is necessary to promote HPV-related knowledge for male and female college students, respectively.

## 1. Introduction

Human papillomavirus (HPV) is a highly infectious virus that is transmitted primarily through sexual intercourse and is one of the most common sexually transmitted infections (STI) in the world [1,2]. HPV infection is a viral infection of the epithelial tissue and, in addition to genital warts, HPV infection can lead to a variety of cancers. Among them, persistent infection with high-risk human papillomavirus (HPV) is the main cause of cervical cancer. Additionally, HPV infection is also associated with several types of cancers, such as anal cancer, head and neck cancer, and oropharyngeal cancer in both men and women [3,4,5]. Some studies have shown that HPV infection causes 60–70% of oropharyngeal squamous cell carcinomas (OPSCCs) [6]. In 2020 alone, China reported 109,741 new cervical cancer cases and 59,060 deaths, corresponding to 18.2% of diagnoses and 17.3% of deaths from cervical cancer worldwide [7], making it the most common cancer of the female reproductive system in China [8]. Men are also at risk for other HPV-related cancers including penile, anal, and oral cancers [9]. Worldwide, HPV infection has posed a significant health threat and disease burden.

Vaccines have been one of the most effective interventions for infectious diseases [10]. The introduction of HPV vaccines in many countries has resulted in a substantial reduction in the prevalence of specific HPV infection, and a recent meta-analysis that included 60 million people showed that HPV vaccination had a substantial impact on the incidence of HPV infection and related diseases [11]. The GlaxoSmithKline 2vHPV vaccine was introduced in mainland China in August 2016, with the 4vHPV vaccine and 9vHPV vaccine being launched in 2017 and 2018, respectively. However, the HPV vaccine is a self-pay vaccine which has not yet been included in China’s national immunization program. The 4vHPV (RMB 798/shot) cost is slightly higher than the 2vHPV (RMB 580/shot). The latest 9vHPV (RMB 1298/shot) is nearly double the price of the 4vHPV. A recent survey showed that only about 3% of women and 1% of men in mainland China have received the HPV vaccine [12], which is clearly a very low rate.

There is now growing evidence that sexually transmitted infections among college students are on the rise and that the college student population is bearing a high burden of sexually transmitted infections. It is clear that they are in the high-risk age group for HPV infection [13,14,15]. In mainland China, the government has not approved HPV vaccination for men, and the risks posed by HPV infection to men have not been given sufficient attention. Most studies on HPV and HPV vaccination attitudes in China currently focus only on women, and few studies have analyzed both male and female knowledge of HPV and HPV vaccine. In addition, college students are at an age where they are able to process new knowledge and decide on whether to receive HPV vaccination independently [16]. Their perceptions, attitudes, and behaviors regarding HPV vaccination are important for their health.

The main objective of this study was therefore to understand the current state of knowledge about HPV and HPV vaccine among male and female college students and the factors that influence vaccination intentions, and to compare the differences between the genders. We hope to provide a scientific basis for policy formulation to improve HPV vaccination rates among college students.

## 2. Materials and Methods

### 2.1. Study Participants

We conducted this cross-sectional study in August 2020 in Wenzhou university town, which has the highest concentration of college students in Wenzhou, with five universities in the region. We calculated the sample size based on the following formula:

(1) $$n=\frac{z_{\alpha /2}^{2}\times p\left(1-p\right)}{d^{2}}$$

where *d* is the permissible error and takes the value of *d* = 0.04 × *p*. *p* is the estimated HPV acceptance rate and based on previous studies [17], we determined that *p* was 73%, taking into account issues such as invalid questionnaires and increasing the sample size by 10%, the final sample size was at least 977. Due to the COVID-19 epidemic, face-to-face investigations were restricted. We generated a link to the online questionnaire via the survey platform (https://www.wjx.cn, accessed on 8 July 2021), then posted the survey link and respondents completed the questionnaire online. We first randomly selected three universities within the university town and then recruited students by convenience sampling. Participants were required to fill in the questionnaire and submit it within a limited period of time. Participants in this study included both males and females. The inclusion criteria were: (1) college students; (2) Chinese citizens, and (3) Chinese reading ability. Participants were informed of the purpose and content of the survey and informed consent was obtained prior to completion. To prevent response bias, participants were guaranteed anonymity and their personal information was kept strictly confidential. In addition, participants received no compensation for their cooperation.

### 2.2. Instruments

Through a review of relevant literature and expert discussion, we designed a questionnaire consisting of four sections, including demographic information, knowledge of HPV infection, knowledge of HPV vaccine and attitudes towards HPV vaccination and reasons for it. In order to quantify the level of knowledge of HPV-related knowledge and HPV vaccine knowledge among college students, we assigned scores to the questions on HPV infection knowledge and vaccine knowledge on the questionnaire, where each question was scored out of 3 or 5 points, with correct answers scored and incorrect answers not scored, out of a total of 55 points. In total, 16 questions were scored out of 44 points for HPV-related knowledge and 4 questions out of 11 points for HPV vaccine knowledge. Details of the questionnaire are attached.

### 2.3. Statistical Analysis

Participants’ demographic characteristics, knowledge of HPV and HPV vaccine, access to knowledge, attitudes to vaccination and related factors were expressed as frequencies and percentages. HPV infection knowledge scores and HPV vaccine knowledge scores were expressed as mean ± standard deviation. Differences between groups were compared by *t*-test and chi-square test, stratified by gender. Attitude towards HPV vaccination was used as the dependent variable, which was categorized as “yes” and “no”, and a multivariable logistic regression analysis was conducted to identify the influencing factors related to the attitude towards HPV vaccination among male and female college students. Statistical significance was assessed by two-tailed tests with α level of 0.05.

## 3. Results

### 3.1. Demographic Characteristics of all Participants

A total of 1035 questionnaires were returned in the end, after excluding 33 invalid questionnaires due to incomplete responses, 1002 questionnaires were included in the final analysis. The demographic characteristics of the study participants are shown in Table 1. The mean age of the participants was 20.52 ± 1.76 years, with 374 males (37.3%) and 628 females (62.7%). Of these, 43 (4.3%) were enrolled in a master’s degree and higher, 617 (61.6%) were medical professionals, and 215 (21.5%) were other members of the household in medical-related occupations.

### 3.2. Knowledge of HPV and HPV Vaccine

In the study, 90.2% and 69.2% of college students had heard of HPV and HPV vaccine respectively. The percentage of males who had heard of HPV and HPV vaccine was 85.3% and 53.7%, respectively, compared to 93.2% and 78.3% of females. The difference was statistically significant (*p* < 0.001). Among college students who had heard of HPV, both males and females mostly learned about HPV through the internet, with 69.4% and 81.6% of college students choosing this option, respectively. Among all college students, the two most desired ways for males and females to learn about HPV were the same: the internet (61.0% vs. 75.6%) and school lectures (60.7% vs. 69.3%), as shown in Table S1 and Table S2.

Table 2 demonstrates the knowledge of college students about HPV and HPV vaccine. College students scored 22.73 ± 9.01 (44 points) and 5.06 ± 3.03 (11 points) for HPV-related knowledge and HPV vaccine knowledge, respectively. In terms of HPV-related knowledge, males and females scored 21.05 ± 9.47 and 23.74 ± 8.58, respectively, with males scoring lower than females and the difference being statistically significant (*p* < 0.001). Specifically, females were more likely than males to know the transmission route of HPV, with a statistically significant difference (*p* = 0.001). As for other questions regarding knowledge of high-risk HPV and HPV-related disease treatment, the scores of males were lower than those of females, with statistically significant differences. In terms of HPV vaccine knowledge, only when asked: “Are you aware of the current status of the HPV vaccine for men today?”, males scored higher than females, with a statistically significant difference (*p* = 0.01). The rest of the HPV vaccine questions were answered better by women than men, with a statistically significant difference.

### 3.3. HPV Vaccination Intention

**Figure 1:**
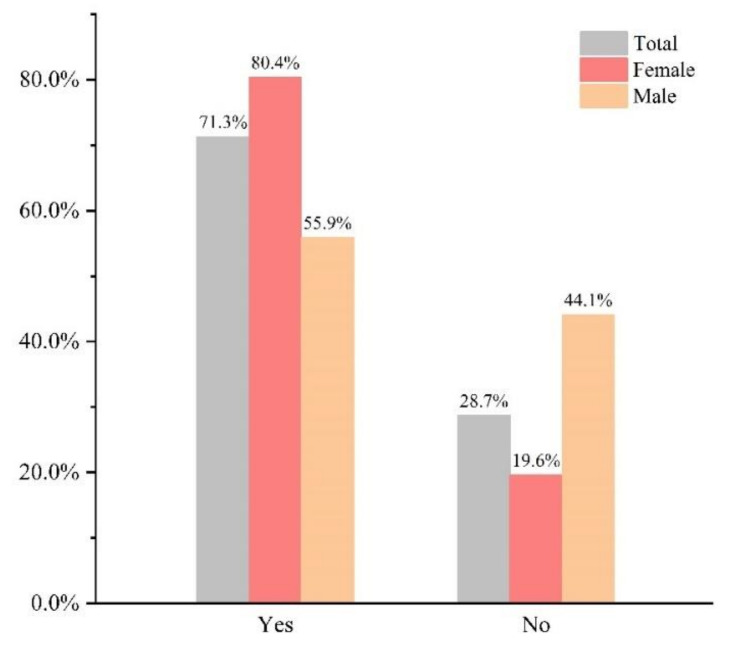
Willingness to vaccinate against HPV. Note: “Yes”: participants were willing to be vaccinated against HPV; “No”: participants were unwilling to be vaccinated against HPV.

**Figure 2:**
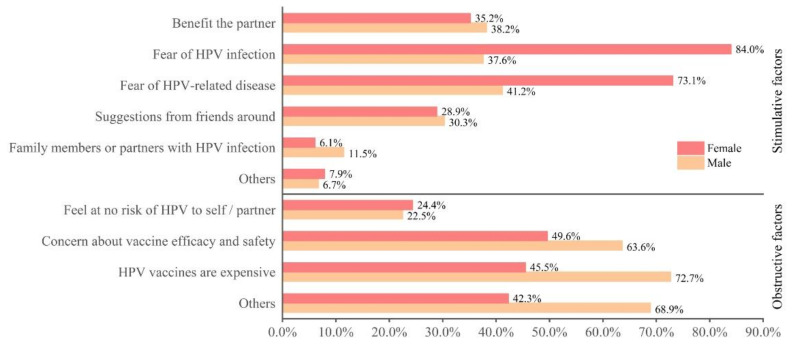
Self-reported facilitators of and barriers to HPV vaccination acceptance. Note: Only participants willing to be vaccinated with HPV answered the question “What are the reasons why you are willing to get HPV vaccination? (multiple choice)” (*n* = 714); only participants who were unwilling to vaccinate with HPV answered the question “What are the reasons that influence you to get HPV vaccination? (multiple choice)” (*n* = 288).

Figure 1 depicts the attitudes of college students towards HPV vaccination, with 714 (71.3%) of them willing to receive HPV vaccination. There was a significant difference between male and female willingness to receive HPV vaccination (55.9% vs. 80.4%, *p <* 0.001). Figure 2 depicts facilitators of and barriers to HPV vaccination acceptance. Among the factors that promoted HPV vaccination, the top three most selected by females were “Fear of HPV infection” (84.0%), “Fear of HPV-related disease” (73.1%), and “Benefit the partner” (35.2%). For males, it was “Fear of HPV-related disease” (41.2%), “Benefit the partner” (38.2%), “Fear of HPV infection” (37.6%). When choosing the factors that prevented them from getting the HPV vaccine, “Concern about vaccine efficacy and safety” was the biggest barrier for males (49.6%), with nearly half of those who did not want to get the HPV vaccine choosing this option. Males cited “HPV vaccines are expensive” as the most important barrier to getting the HPV vaccine (72.7%). When asked “Which of the followings is most likely to prompt your own vaccination?”, 73.4% of females chose “Vaccine price reduced to acceptable range”, unlike females, “Doctor’s advice” was the most frequently chosen option by males (57.0%), as shown in Table S3.

### 3.4. Factors Associated with the Willingness to Receive HPV Vaccination

A multivariate logistic regression analysis of the factors associated with college students’ willingness to receive HPV vaccination are shown in Table 3. The male gender (aOR = 0.388, 95% CI 0.287–0.527) was a significant predictor of reluctance to receive HPV vaccine, while higher HPV infection knowledge score (aOR = 1.706, 95% CI: 1.225–2.378) and higher HPV vaccine knowledge score (aOR = 2.329, 95% CI: 1.644–3.299) were significant predictors of willingness to receive HPV vaccine. Analysis stratified by gender showed different results for males and females. Having heard of the HPV vaccine and a higher HPV vaccine knowledge score (aOR = 3.486, 95% CI: 2.122–5.729) were significant predictors of willingness to receive the HPV vaccine among female college students, whereas high willingness among males was only associated with a higher HPV-related knowledge score (aOR = 1.868, 95% CI: 1.154–3.022), as shown in Table 4.

## 4. Discussion

In this study, we found that the knowledge of HPV and HPV vaccine among college students in Wenzhou was at a medium to low level, even though awareness of HPV and HPV vaccine was high. The overall knowledge level of males is lower than that of females. In addition, for male college students, providing more knowledge about HPV infection is helpful to promote their willingness to vaccinate. The main findings of this study may help to provide effective educational guidance and interventions for improving the vaccination rate of the target population in the future.

The HPV vaccine does not prevent people who are already infected with HPV from developing cervical cancer, so the highest efficacy of the HPV vaccine is when it is administered before sexual debut. In fact, many Western countries have school-based vaccination programs for 12–13 years old boys and girls [18]. In the UK, the NHS vaccination program to prevent cervical cancer has so far stopped thousands of women from developing the disease and experiencing pre-cancerous changes to cells [19]. However, we believe that it is valuable to investigate HPV vaccination among Chinese college students. Influenced by traditional Chinese culture, Chinese college students are relatively conservative in their thinking, the situation of college student sexual behavior in China is different from that in Europe. In Guangzhou, a developed city in China, a survey of college students showed that a majority (85.2%) of students claimed that they had never engaged in sexual activity [20], while a Greek survey of adolescents aged 15–21 found that 48.0% of students answered that they had been sexually active [21]. Furthermore, college students not only fall in the age group at high risk for HPV infection but also in childbearing age, the decision to receive the vaccine or not has an impact on their risk of acquiring HPV, which can also be passed on to the next generation if HPV is present during delivery. Moreover, according to a meta-analysis in 2019 [11], HPV-related endpoints such as HPV16, HPV18, anogenital warts, and cervical intraepithelial neoplasia grade 2+ (CIN2+) decreased significantly among women aged 20–24 years following HPV vaccination. For the above reasons, it is very meaningful to promote HPV vaccination among Chinese college students.

Since the development of the HPV vaccine, numerous research articles have appeared in China on the knowledge, attitudes, and behavioral tendencies of college students [22]. According to the survey results, the awareness of HPV and HPV vaccine was higher among college students in the current study compared to previous studies of Chinese college students. In a 2013 study, only 10.3% and 5.4% of college students had heard of HPV and HPV vaccine, respectively [23], and in a 2014 survey of Chinese college students’ awareness of HPV and HPV vaccine, the figures were only 14.3% and 8.1% [24]. After the HPV vaccine was approved in China in 2016, Chinese college students began to gradually become aware of HPV and HPV vaccine. A survey in Changsha in 2017 showed that the awareness rate of college students about HPV vaccine was 32.4% [25] and a study conducted in Beijing in 2018 found that the awareness rate of college students about HPV and HPV vaccine was 76.5% and 72.6%, respectively [26]. The increased publicity by the authorities in recent years and the widespread availability of HPV vaccination in China may explain the high awareness of HPV and HPV vaccine in this study. In addition, as the majority of participants had a medical background, that may have contributed to higher knowledge on HPV. Medical students were reported to be more likely to have heard of HPV and to have a higher level of knowledge than non-medical students [27].

From the results of this study, the scores of college students’ knowledge of HPV and HPV vaccine were 22.73 ± 9.01 (44 points) and 5.06 ± 3.03 (11 points), respectively, which are at a low to moderate level, indicating that although the awareness rate has increased, college students’ knowledge of HPV and HPV vaccine is still low. In our study population, a large proportion of college students, both males and females, were unaware that HPV can infect men, and that more education intervention targeted at men is needed. In answering the question on HPV and diseases associated with high-risk HPV infection, males performed worse than females, although both scores were low, indicating to some extent the lack of knowledge of HPV infection among college students, especially among male students. The fact that the HPV vaccine is not approved for use in males in mainland China and that HPV infection is mainly associated with cervical cancer in females has contributed to this phenomenon. In a survey conducted in 2015, there was a need to improve the education of young males about HPV infection, its associated diseases, and the benefits of the vaccination [26]. Furthermore, the results showed that college students of both sexes were not very clear about the basis of diagnosis of HPV infection, which may delay early consultation of those with suspected infection. When asked about HPV vaccine-related issues, females in general showed a higher level of knowledge than males, and females were more aware than males of the period of HPV vaccination and the benefits of the vaccination, in line with the findings of some previous national and international studies [27,28,29]. We found that the internet was the most important source of information for college students, both males and females, who were aware of HPV. In addition, the internet and popular science lectures were the most chosen avenues of expectation for all to learn about HPV, which is similar to the results of international studies [30,31]. Healthcare providers have been shown to play a pivotal role in promoting vaccination in different settings and other risk groups [32], and schools should strengthen their collaboration with health care providers, which may be an effective measure to increase HPV knowledge among college students.

Proportion of college students with the intention to vaccinate in several studies in China ranged from 33 to 70% [20,23,24,33]. When the HPV vaccine was not yet available in mainland China, studies in 2012 and 2013 showed a high intention to vaccinate among college students at 70% and 70.6%, respectively [23,24]. However, in the two studies in 2018 and 2019, this figure was only 44.4% and 33.7%, respectively [20,33]. This may be due to the vaccine scandal that took place in China in 2018, when a large number of substandard vaccines reached the market. A study demonstrated that public confidence was significantly affected by the vaccine scandal, particularly for vaccine producers and drug regulators [34]. In contrast, the higher vaccination intention in this study may be due to the fact that Wenzhou is located in the eastern region of China, and a recent study showed that college students in the eastern region of mainland China are more willing to get the HPV vaccine compared to the western region [35]. In addition to this, the COVID-19 pandemic in the first half of 2020 has promoted concern for people’s own health, and the results of several studies have shown that people’s fear of COVID-19 may promote preventive health behaviors, thus acting as a form of protection [36,37].

In exploring factors influencing attitudes towards HPV vaccination, our findings were similar to those of some other studies [24,33]. The findings of the study suggest that fear of HPV infection and the perceived benefits to be gained from HPV vaccination were facilitators of HPV vaccination among college students. At the same time, the lack of information about HPV vaccination, as well as concerns about side effects and potential safety issues, discouraged college students from receiving the HPV vaccine, a finding that is consistent with national and international studies [27,38]. Male college students were more concerned about the price of the HPV vaccine. In addition, the lower price of the HPV vaccine was also an important facilitator of HPV vaccination among female college students. In mainland China, medical insurance does not cover the HPV vaccine and requires individuals to pay for the vaccination, which costs between RMB 1800 and 4000 for three shots. Although differences in perceptions of the cost of vaccination may be related to socioeconomic status, this may be an important factor in deciding whether or not to receive HPV vaccine [33]. In addition to providing HPV knowledge, doctors can also affect college students’ HPV vaccination attitude. Logistic regression results show that gender differences influence willingness to receive the vaccine, and we found that female college students who had heard of the HPV vaccine had a higher willingness to receive the vaccine, while there was no effect for males, possibly because the term “cervical cancer vaccine” is often used in the marketing of the HPV vaccine, one might assume that it is not relevant to men. More specifically, the higher level of HPV vaccine knowledge among female college students was an important contributing factor to their HPV vaccination, while male participants’ knowledge about HPV infection may have had a greater impact on them. This result suggests that targeted assistance could be provided to both male and female college students, focusing on the role and knowledge of the HPV vaccine for female students, and more knowledge about HPV infection for males, in order to improve overall HPV vaccination rates among college students.

Studies in several countries have shown that extending the current HPV vaccination program to the male population is a highly cost-effective preventive intervention that can better prevent diseases such as anogenital cancer, a proportion of head and neck cancer, cancer precursors, and genital warts in both men and women [39,40]. High HPV vaccine coverage in both sexes would help achieve herd immunity and largely reduce HPV, but it is currently difficult to include the HPV vaccine in China’s national immunization program. China is still a developing country with a large population. In addition, China is not a GAVI (The Global Alliance for Vaccines and Immunization) supported country and thus may not receive extensive financial assistance to maintain the HPV vaccine supply [20]. However, the Chinese government is accelerating free HPV vaccination and actively seeking cooperation with organizations such as GAVI. From 2020, some cities in China have already started to gradually introduce free HPV vaccination among girls of school age, exploring suitable experiences for the promotion of free HPV vaccination. Given China’s vast geography and significant regional differences in economic development and disease burden [41], we believe it is challenging to address the issue of free HPV vaccination in China with one model, and we need to actively explore more different models to promote free HPV vaccination in China. The first Chinese domestic HPV vaccine against HPV 16 and 18 (Cecolin, Innovax, Xiamen, China) was licensed by the Chinese Food and Drug Administration on 31 December 2019 and approved for a two-dose schedule for girls aged 9−14 years. The new vaccine Cecolin is priced at RMB 329 per dose (about half of the price of Cervarix) and has similar efficacy to Cervarix, providing an unprecedented opportunity for China [42].

It is necessary for government and universities to take targeted measures such as establish a national financial subsidy, increasing health education related to HPV infection in the school curriculum, and promoting knowledge about HPV and HPV vaccine through the internet and social media. Vaccine health education and the development of healthy behaviors among college students depend not only on the support of the government, medical institutions, and schools, but also on the increased awareness of college students to prevent HPV infection. Only by recognizing the seriousness and consequences of HPV infection and the effectiveness of the HPV vaccine can the overall HPV vaccination rate among college students be increased.

There are several limitations to this study. Firstly, this study used self-reported data collected using a questionnaire and may be subject to self-report bias and recall bias. Secondly, due to the cross-sectional nature of the study, strong causal relationships cannot be provided and caution should be exercised in interpreting the influences. Finally, as the majority of college students in this study had medical backgrounds, the study population was limited in its representativeness.

## 5. Conclusions

This study shows that college students of both sexes in Wenzhou have a medium level of willingness to accept HPV vaccine, a medium to low level of knowledge about HPV and HPV vaccine, and a lower overall level of knowledge among males than females. The low level of knowledge about HPV and HPV vaccine and the high price of the vaccine are the main barriers to HPV vaccination among college students. For male college students, providing more knowledge about HPV infection is helpful to promote their willingness to vaccinate. Strengthening health education on HPV-related knowledge and lowering the price of the vaccine would help promote the HPV vaccine among the college student population.

## Figures and Tables

**Table 1:** Demographic characteristics of study participants (*n* = 1002).

Characteristics	Frequency (*n*)	Percent (%)
Gender		
Female	628	62.7
Male	374	37.3
Age (years)		
<20 years	223	22.3
20–23 years	686	68.5
≥23 years	93	9.3
Reading record of formal schooling		
Bachelor’s degree	959	95.7
Master’s degree and higher	43	4.3
Major		
Non-medical	385	38.4
Medical	617	61.6
Family members engaged in medical related occupations		
No	787	78.5
Yes	215	21.5

**Table 2:** College students’ knowledge (based on scores) of HPV (out of 44 points) and HPV vaccination (out of 11 points).

	Questions (Points)	Total	Female	Male	*p*
HPV-related knowledge	Do you know that men can also be infected with HPV? (3)	1.23 ± 0.86	1.24 ± 0.82	1.23 ± 0.93	0.842
	What are the ways of transmission of HPV do you know? (3)	1.52 ± 1.11	1.61 ± 1.09	1.36 ± 1.13	0.001
	Do you think men and women with HPV infection need to be treated? (3)	2.97 ± 0.22	2.97 ± 0.22	2.97 ± 0.21	0.987
	Which of the following diseases do you think are associated with HPV infection? (5)	2.89 ± 1.47	3.09 ± 1.41	2.57 ± 1.51	0.000
	Which of the following male diseases do you think are caused by HPV? (3)	0.99 ± 0.99	1.01 ± 0.99	0.95 ± 0.99	0.396
	What do you think is the difference between high-risk HPV and low-risk HPV? (3)	2.00 ± 1.42	2.14 ± 1.36	1.76 ± 1.48	0.000
	Which diseases do you think are caused by high-risk HPV? (4)	1.72 ± 1.51	1.84 ± 1.51	1.51 ± 1.49	0.001
	Do you know anything about transient HPV infection? (3)	1.01 ± 0.88	1.04 ± 0.87	0.97 ± 0.92	0.208
	Do you know about the self-limiting nature of HPV? (3)	1.09 ± 0.93	1.10 ± 0.91	1.08 ± 0.97	0.798
	Do you think there is cross infection between sexual partners? (3)	2.21 ± 1.32	2.35 ± 1.24	1.97 ± 1.43	0.000
	Do you know the basis for the diagnosis of HPV? (3)	0.66 ± 1.24	0.71 ± 1.28	0.56 ± 1.17	0.058
	Do you think men with HPV infection can be cured? (3)	2.11 ± 1.37	2.16 ± 1.35	2.03 ± 1.41	0.151
	Which of the following treatments do you consider to be effective? (5)	2.34 ± 1.88	2.49 ± 1.91	2.10 ± 1.81	0.001
	Total (44)	22.73 ± 9.01	23.74 ± 8.58	21.05 ± 9.47	0.000
HPV vaccine knowledge	Which group of people do you think should be vaccinated against HPV? (3)	1.25 ± 1.48	1.52 ± 1.50	0.79 ± 1.32	0.000
	Do you know the current status of the HPV vaccine for men today? (3)	0.56 ± 1.17	0.49 ± 1.11	0.69 ± 1.26	0.010
	What do you think is the best time to get HPV vaccination? (3)	2.01 ± 1.41	2.22 ± 1.32	1.65 ± 1.49	0.000
	Which of the following diseases do you think HPV vaccination can prevent? (2)	1.24 ± 0.90	1.33 ± 0.88	1.09 ± 0.92	0.000
	Total (11)	5.06 ± 3.03	5.56 ± 2.89	4.22 ± 3.07	0.000

**Table 3:** Factors associated with the willingness to receive HPV vaccination.

Variable	OR (95% CI)	aOR (95% CI)
Gender		
Female	ref	ref
Male	0.309 (0.232–0.410) ***	0.388 (0.287–0.527) ***
Reading record of formal schooling		
Bachelor’s degree	ref	ref
Master’s degree and higher	2.131 (0.937–4.847)	2.513 (0.866–5.353)
Major		
Non-medical	ref	ref
Medical	1.312 (0.993–1.734)	0.983 (0.707–1.368)
Family members engaged in medical related occupations		
No	ref	ref
Yes	1.343 (0.949–1.900)	1.354 (0.927–1.976)
Heard of HPV		
No	ref	ref
Yes	3.514 (2.297–5.375) ***	1.533 (0.932–2.524)
Heard of the HPV vaccine		
No	ref	ref
Yes	3.186 (2.387–4.252) ***	1.397 (0.972–2.008)
HPV infection knowledge score		
Low (≤22)	ref	ref
High (>22)	2.615 (1.971–3.469) ***	1.706 (1.225–2.378) **
HPV vaccine knowledge score		
Low (≤5)	ref	ref
High (>5)	3.612 (2.634–4.952) ***	2.329 (1.644–3.299) ***

Note: Variables in univariate logistic regression models eventually entered the multivariable logistic regression model. HPV, human papillomavirus; OR, odds ratio; aOR, adjusted odds ratio; CI, confidence interval. “**” indicates *p* < 0.01, “***” indicates *p* < 0.001.

**Table 4:** Factors associated with the willingness to receive HPV vaccination by gender.

Variable	Female		Male	
	OR (95% CI)	aOR (95% CI)	OR (95% CI)	aOR (95% CI)
Reading record of formal schooling				
Bachelor’s degree	ref	ref	ref	ref
Master’s degree and higher	3.284 (0.769–14.027)	2.683 (0.571–12.617)	1.608 (0.539–4.800)	1.960 (0.610–6.303)
Major				
Non-medical	ref	ref	ref	ref
Medical	1.236 (0.827–1.847)	1.037 (0.657–1.637)	1.360 (0.897–2.063)	1.046 (0.644–1.700)
Family members engaged in medical related occupations				
No	ref	ref	ref	ref
Yes	1.220 (0.734–2.028)	1.224 (0.705–2.124)	1.687 (1.024–2.780) *	1.603 (0.956–2.688)
Heard of HPV				
No	ref	ref	ref	ref
Yes	5.020 (2.660–9.475) ***	1.560 (0.736–3.309)	1.946 (1.089–3.476) *	1.364 (0.714–2.607)
Heard of the HPV vaccine				
No	ref	ref	ref	ref
Yes	4.440 (2.895–6.811) ***	2.269 (1.353–3.805) **	1.527 (1.012–2.303) *	0.927 (0.557–1.542)
HPV infection knowledge score				
Low (≤22)	ref	ref	ref	ref
High (>22)	2.590 (1.730–3.878) ***	1.574 (0.983–2.522)	2.188 (1.437–3.331) ***	1.868 (1.154–3.022) *
HPV vaccine knowledge score				
Low (≤5)	ref	ref	ref	ref
High (>5)	4.917 (3.064–7.891) ***	3.486 (2.122–5.729) *	1.990 (1.259–3.146) **	1.520 (0.901–2.565)

Note: Variables in univariate logistic regression models eventually entered the multivariable logistic regression model. HPV, human papillomavirus; OR, odds ratio; aOR, adjusted odds ratio; CI, confidence interval. “*” indicates *p* < 0.05, “**” indicates *p* < 0.01, “***” indicates *p* < 0.001.

## Data Availability

The data that support the findings of this study is available, you can request the data directly from the authors. E-mail: andrologywzg@wmu.edu.cn.

## Supplementary Materials

The following are available online at https://www.mdpi.com/article/10.3390/vaccines10010010/s1, Table S1: Manners to HPV understanding (*n* = 904), Table S2: Desired manners to gain further insight into HPV (*n* = 1002), Table S3: Self-perceived factors that promote HPV vaccination (*n* = 1002).
