# Supplementary material for: Gender Differences in Knowledge and Attitude towards HPV and HPV Vaccine among College Students in Wenzhou, China
Source: Vaccines (Basel). 2021 Dec 22;10(1):10. doi: 10.3390/vaccines10010010 (PMC8779512; doi:10.3390/vaccines10010010)
Supplement: Supplementary file 1 [file vaccines-10-00010-s001.zip › vaccines-1440895-supplementary.pdf]

Table S1. Manners to HPV understanding (n = 904).

Manners	Total	Female	Male
School science lectures	325(45.5%)	225(44.6%)	100(47.8%)
Internet	557(78.0%)	412(81.6%)	145(69.4%)
Physician consultation	125(17.5%)	89 (17.6%)	36(17.2%)
Newspapers and magazines	220(30.8%)	159(31.5%)	61(29.2%)
Others	214(30.0%)	150(29.7%)	64(30.6%)

Note: A total of 904 participants have heard of HPV, and only they can answer Q7 " From which of the following sources did you obtain information about HPV? (Multiple choice) " .

Table S2. Desired manners to gain further insight into HPV (n = 1002).

Manners	Total	Female	Male
School science lectures	662(66.1%)	435(69.3%)	227(60.7%)
Internet	703(70.2%)	475(75.6%)	228(61.0%)
Social activities	575(57.4%)	390(62.1%)	185(49.5%)
Newspapers and magazines	402(40.1%)	269(42.8%)	133(35.6%)
Others	190(19.0%)	99(15.8%)	91(24.3%)

Table S3. Self-perceived factors that promote HPV vaccination (n = 1002).

Factors	Total	Female	Male
Doctor's advice	572(57.1%)	359(57.2%)	213(57.0%)
Free HPV vaccination	584(58.3%)	372(59.2%)	212(56.7%)
Partner has a history of HPV-related disease	258(25.7%)	139(22.1%)	119(31.8%)
Most friends are willing to get vaccinated	441(44.0%)	292(46.5%)	149(39.8%)
Vaccine price reduced to acceptable range	655(65.4%)	461(73.4%)	194(51.9%)
Others	143(14.3%)	80(12.7%)	63(16.8%)
